# A Theory-Based, Multidisciplinary Approach to Cocreate a Patient-Centric Digital Solution to Enhance Perioperative Health Outcomes Among Colorectal Cancer Patients and Their Family Caregivers: Development and Evaluation Study

**DOI:** 10.2196/31917

**Published:** 2021-12-07

**Authors:** Su Wei Wan, Choon Seng Chong, Ee-Lin Toh, Siew Hoon Lim, Carol TT Loi, Yuen Foong Henry Lew, Matthew Chin Heng Chua, Xin Pei Jee, Guangyu Liu, Lixia Zhu, Minna Pikkarainen, Hong-Gu He

**Affiliations:** 1 Alice Lee Centre for Nursing Studies National University of Singapore Singapore Singapore; 2 National University Health System Singapore Singapore; 3 Division of Colorectal Surgery and Surgical Oncology, Department of Surgery, National University Cancer Institute National University Hospital Singapore Singapore; 4 Yong Loo Lin School of Medicine National University of Singapore Singapore Singapore; 5 Department of Colorectal Surgery Singapore General Hospital Singapore Singapore; 6 Duke-NUS Medical School Singapore Singapore; 7 Division of Nursing Singapore General Hospital Singapore Singapore; 8 Centre of Psychosocial Support Singapore Red Cross Academy Singapore Singapore; 9 Department of Psychology School of Humanities and Behavioural Science Singapore University of Social Sciences Singapore Singapore; 10 Medical and Cybernetics Systems Institute of Systems Science National University of Singapore Singapore Singapore; 11 Division of Colorectal Surgery, Department of Nursing National University Hospital Singapore Singapore; 12 Faculty of Health Sciences and Faculty of Technology, Art and Design Oslomet - Oslo Metropolitan University Oslo Norway; 13 Department of Electrical Engineering Chalmers University of Technology Gothenburg Sweden; 14 Faculty of Medicine and Oulu Business School University of Oulu Oulu Finland

**Keywords:** colorectal cancer, digital solutions, mobile health, psychosocial, mHealth, smartphone app, mobile phone app

## Abstract

**Background:**

Elective colorectal cancer (CRC) surgeries offer enhanced surgical outcomes but demand high self-efficacy in prehabilitation and competency in self-care and disease management postsurgery. Conventional strategies to meet perioperative needs have not been pragmatic, and there remains a pressing need for novel technologies that could improve health outcomes.

**Objective:**

The aim of this paper was to describe the development of a smartphone-based interactive CRC self-management enhancement psychosocial program (iCanManage) in order to improve health outcomes among patients who undergo elective CRC surgeries and their family caregivers.

**Methods:**

A multidisciplinary international team comprising physicians, specialist nurses, a psychologist, software engineers, academic researchers, cancer survivors, patient ambassadors, and ostomy care medical equipment suppliers was formed to facilitate the development of this patient-centric digital solution. The process occurred in several stages: (1) review of current practice through clinic visits and on-site observations; (2) review of literature and findings from preliminary studies; (3) content development grounded in an underpinning theory; (4) integration of support services; and (5) optimizing user experience through improving interface aesthetics and customization. In our study, 5 participants with CRC performed preliminary assessments on the quality of the developed solution using the 20-item user version of the Mobile App Rating Scale (uMARS), which had good psychometric properties.

**Results:**

Based on the collected uMARS data, the smartphone app was rated highly for functionality, aesthetics, information quality, and perceived impact, and moderately for engagement and subjective quality. Several limiting factors such as poor agility in the adoption of digital technology and low eHealth literacy were identified despite efforts to promote engagement and ensure ease of use of the mobile app. To overcome such barriers, additional app-training sessions, an instruction manual, and regular telephone calls will be incorporated into the iCanManage program during the trial period.

**Conclusions:**

This form of multidisciplinary collaboration is advantageous as it can potentially streamline existing care paths and allow the delivery of more holistic care to the CRC population during the perioperative period. Should the program be found to be effective and sustainable, hospitals adopting this digital solution may achieve better resource allocation and reduce overall health care costs in the long run.

**Trial Registration:**

ClinicalTrials.gov NCT04159363; https://clinicaltrials.gov/ct2/show/NCT04159363

## Introduction

Colorectal cancer (CRC) is known to be one of the leading causes of cancer-related morbidity and mortality among both men and women worldwide [1]. It will continue to be a global threat because of its profound impact on societies and public health [2]. Surgical tumor resection is the primary treatment for colorectal cancer, and as worldwide incidences continue to surge, the corresponding demand for elective surgeries is expected to rise as well [3]. The period leading up to and following an operation can be exceptionally sensitive and challenging for some patients, given the state of psychological vulnerability they are in, the debilitating symptoms, and the lifestyle changes they concurrently contend with [4]. Under the current enhanced recovery program or “fast-track surgery,” much of the preoperative physiologic optimization occurs outside of hospital [5,6], and shortened hospitalization stays further mandate or pressurize CRC survivors to be independent and competent in self-care and disease management postdischarge [7,8]. Despite the introduction of various interventions in recent years, frequent, unwarranted hospital readmissions resulting from postoperative complications (eg, surgical site infections, intestinal obstruction, bleeding, and ostomy malfunction) imply that moderate self-efficacy levels reported among CRC survivors are suboptimal [9-12]. Furthermore, informal caregivers have been reported to lack confidence and have inadequate training in managing their care recipients’ bowel problems, pain, fatigue, medications, and other symptoms [13]. These combined findings justify the need for more comprehensive and targeted measures. Therefore, this paper aimed to describe the development of a smartphone-based interactive CRC self-management psychosocial program (iCanManage) in order to improve perioperative health outcomes among patients with CRC undergoing elective CRC surgeries and their family caregivers.

## Methods

### Overview

The initial stage involved the establishment of a multidisciplinary international team by the study’s principal investigator. Following the recruitment of a few academics, clinicians from the 2 largest acute hospitals in Singapore, ostomy care appliance specialists, as well as software engineers from a renowned Finnish company were approached. Meetings were organized to assemble all parties to brainstorm for ideas and kick-start the construction and mapping of timelines delineating critical milestones to be reached. The roles and the respective responsibilities were delegated (Table 1), and a reporting cascade was put in place to facilitate consistency and consensus throughout the development of the patient-centric digital solution. The appointed project manager assisted with the arrangement of all team-based activities and timely documentation of the project’s progress.

**Table 1:** Multidisciplinary team members and roles.

Key personnel	Partner country	Role in the development process
Principal investigator	Singapore	Conceptualize and supervise the iCanManage development and trial process
Project manager	Singapore	Coordinate communication among collaboration partners and key stakeholders
Medical doctors	Singapore	Provide content expertise for the creation of surgery care paths
Specialist nurses	Singapore	Provide content expertise on perioperative assessments and counseling, symptom management, and self-care
Psychologist	Singapore	Provide content expertise to develop, structure, and arrange mindfulness-based practices and activities Provide formal psychological support
Software engineers	Finland and Singapore	Design digital solution, input content within care path, and maximize functionality and customization of the software interface
Academic researchers	Singapore and Finland	Collect field data, expert opinion, and public feedback before trial testing
Cancer survivors and patient ambassadors	Singapore	Provide informal peer support through sharing of personal experiences, identification of common challenges, and coping strategies. Provide information on external support services
Cancer institute and society	Singapore	Provision of patient education pamphlets, brochures, and booklets
Ostomy care medical equipment suppliers	Denmark (Coloplast) and United States (ConvaTec)	Provide multimedia on ostomy care, instructional guidelines, and practical tips on lifestyle adjustment after stoma creation

### Review of Current Clinical Practice

To grasp a better understanding and to identify gaps in the current perioperative workflow of local hospital outpatient settings, the academic study team members conducted multiple clinic visits and on-site observations. Physical examinations, clinical procedures, care plan discussions, as well as types of queries and concerns raised by the patients and their accompanied caregivers during medical consultation sessions, were manually recorded. Preoperative assessments and instructions, educational reference materials, and relevant counseling advice provided by specialist nurses based on the patients’ physiological status were also collected and documented. Besides the aforementioned, some study team members attended in-house peer support sessions, organized by patient ambassadors, and talks involving ostomy awareness to get a sense of the topics shared and activities availed for participation. These public events enabled conversations exploring patients with CRC and their caregivers’ struggles, needs, satisfaction with care, and support preferences. Information about the referral process for formal psychosocial support was sought for knowledge about equity of access to such resources.

Through these reviewed processes, a few critical issues were identified. Apart from the occasional linguistic mismatch, a handful of patients, in particular those who attended the preoperative counseling alone, expressed confusion at the information provided by the specialist nurses. Caregivers who were unavailable for the face-to-face teaching were often left at a loss, resulting in multiple calls to the hospital for clarifications. Moreover, nurses themselves opined that the session was too overwhelming for those still fragile from receiving their diagnosis. They noticed that many of the seniors with CRC had difficulty retaining the given instructions and misplaced the reference materials provided. It was a constant challenge for 1 of the hospitals to ensure that the patients and their caregivers were competent with stoma care within the short hospitalization stay before discharge. Furthermore, peer-sharing sessions came to a halt as soon as COVID-19 emerged, further depriving the patients of the already minimal psychosocial support. These gaps highlighted major deficiencies in current practice and triggered this initiative to involve novel technologies to address the complex needs of this population.

### Review of Literature and Findings From Preliminary Studies

The existing empirical evidence postulates that individuals with higher self-efficacy are more likely to be able to overcome illness-related challenges. Within the CRC population, substantial literature has reported positive associations between self-efficacy, adjustment to colostomy, lifestyle adaptation, symptom coping, social support, physical health, psychological well-being, and quality of life [8,14-19]. One study found presurgery self-efficacy, anxiety, and depression to be important predictors of one’s recovery [12], and this is a significant finding because of the interdependent relationships between patients and their caregivers’ self-efficacy as well as physical and mental health [20,21].

For CRC patients in particular, psychosocial care plays a subsidiary role in biomedical treatment where its positive effects on multiple mental health outcomes and quality of life have been verified in several reviews [22-24]. However, as COVID-19 continues to persist, conventional face-to-face perioperative counseling by health care professionals has become increasingly challenging. Access to psychosocial support is limited and confined to those deemed to be at risk of significant psychological morbidity. This affects up to approximately 160 patients, who undergo elective colorectal resections annually in 1 local health care institution alone [25], and aggravates the mental distress already prevalent within this population [26]. Moreover, a preliminary study found great appetite for easily retrievable, in-depth, credible information about treatment and interest in the use of health technologies with embedded multimedia [27]. Such indications call for the need to review existing care delivery models and consider possible shifts toward the adoption of telemedicine.

### Content Development Based on Self-efficacy Theory and Literature

**Figure 1:**
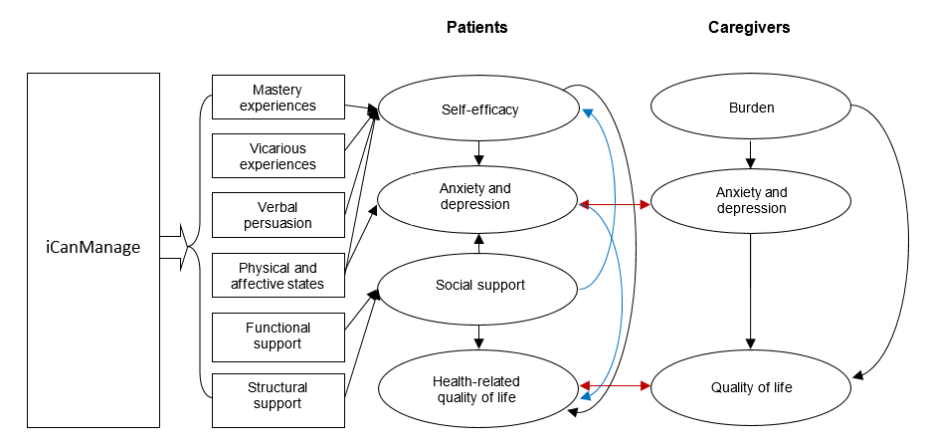
Conceptual framework for the iCanManage program.

Having put together all the gathered information, we developed the iCanManage program based on the self-efficacy theory by Bandura [28] and the interrelationships between self-efficacy, anxiety, depression, social support, burden, and quality of life, as previously described (Figure 1). This theory rides on the core assumption that the likelihood of a person engaging in a behavior depends on their conviction that they are able to successfully execute the course of action required to produce the intended outcome. Once the behavior has been adopted, the individual estimates and evaluates whether the given behavior leads to certain outcomes. In this theory, it is postulated that self-efficacy stems from four sources, namely mastery experiences, vicarious experiences, verbal persuasion, and emotional and physiological states, which we will describe in the following section.

#### Mastery Experiences

Mastery experiences are especially influential, and they accumulate from persistent and sustained efforts in achieving performance success and overcoming failures, obstacles, and adversity. The self-efficacy developed through mastery experiences refers to resilience and the ability to quickly rebound when faced with setbacks.

#### Vicarious Experiences

Vicarious experiences are gained from assimilation to the successes and failures of social models. Through the observation of similar individuals who succeed, people come to believe that they too are capable of succeeding. Conversely, observing similar individuals who fail despite their high effort will lower one’s expectations and prevent self-criticism.

#### Verbal Persuasion

Verbal persuasion from social interactions can also enhance one’s perceived self-efficacy. Through the use of positive words, people’s belief that they have the capacity to succeed is strengthened, and they are motivated to mobilize greater effort to sustain or confront the challenges that come their way.

#### Physical and Affective States

Self-efficacy is also influenced by how an individual views and understands emotional and physical reactions, including stress, tension, fatigue, and mood states, to be a reflection of their performance abilities. By learning how to regulate their responses when encountering difficult and challenging situations, one can cultivate a greater locus of control and develop self-efficacy.

CRC surgeries, unlike those for other cancer types, bring about many physical and lifestyle changes that require the acquisition of new behavior patterns [29]. Yet, successful adaptation and adjustment usually follow a feedback loop involving response consequences and predictive cues, which are largely influenced by the individual’s cognitive processes and perceived locus of control [28]. Bandura’s self-efficacy theory has been widely used within the cancer population to guide intervention development, where significant positive effects on numerous health outcomes were reported [19,30]. Knowing how demanding and unpredictable the course of this illness is, it became imperative to design the iCanManage program to empower our trial participants by enabling access to the four sources of self-efficacy. By doing so, the participants will have greater preparedness for surgery, which may benefit recovery after surgery [31]. At the same time, they will also be better positioned to respond and withstand the ordeal in the long run [14]. A description of how the contents were designed based on this theory is presented in Multimedia Appendix 1.

### Integration of Support Services

Apart from formal professional support, tangible and practical help are also essential for sustained adaptive coping, especially after an individual is discharged from the health care institution and reintegrates back into the community [4,32]. Hence, cancer survivors and patient ambassadors of external support groups (eg, Ostomy Association of Singapore, Singapore Cancer Society, and Patient Advocacy Network) were invited to share their personal experiences in the form of videos to relay useful messages on resuming activities of daily living while at the same time providing companionship throughout the receiving patient’s cancer journey. These videos were later embedded within the digital solution.

In Singapore, a majority of patients with CRC fitted with a stoma (ie, ileostomy or colostomy) obtain their ostomy care appliances from Coloplast (Coloplast A/S) and ConvaTec (ConvaTec Group plc). For many years, these 2 companies from Denmark and the United States, respectively, have been committed to providing advanced therapies for wound, ostomy, and continence care [33,34]. To ensure our trial participants are able to manage their stoma and quickly familiarize themselves with the use of different ostomy products, partnerships with these companies were built to borrow educational multimedia for dissemination through the digital solution. This collaboration also opened a platform for the trial participants to connect with the product suppliers and ostomy care specialists directly.

### Optimizing User Experience

Once the final version of the proposed contents was ready, software engineers from Buddy Healthcare were engaged to input all materials into the created digital solution known as BuddyCare. Buddy Healthcare is known for its pioneering efforts in designing award-winning automated care pathways for improved care coordination and patient engagement [35]. To date, they have over 200 care pathway templates for elective surgeries in use by hospitals across various continents (Europe, United States, and Asia Pacific), where positive results have been attained, including significant reductions in preoperative phone calls and the time spent on counseling, as well as high recommendation ratings from health care professionals who have subscribed to the platform.

To ensure seamless navigation and optimal user experience, iterative processes of trial and error took place to format and arrange the content layout to be displayed. During these evaluation sessions, some notable points were raised: (1) ensuring a sizeable but digestible amount of information load; (2) modulating the appropriate time spent and the daily app usage; (3) using elderly friendly fonts, text size, and colors; (4) using icons to increase the visibility of new instructions; and (5) controlling the frequency of reminders, alerts, and notifications.

After the smartphone-based digital solution was fine-tuned, it was formally implemented as part of the intervention component of a multicenter randomized controlled trial (NCT04159363) conducted at 2 large-scale acute tertiary hospitals. At present, the full-scale randomized controlled trial is ongoing, and the data are insufficient for analysis. However, apart from our main study outcomes, preliminary assessments and feedback on BuddyCare were solicited using the Mobile App Rating Scale: User Version (uMARS). This 20-item instrument was developed by Stoyanov and colleagues to measure the quality of mHealth apps across the 5 subscales of engagement (5 items), functionality (4 items), aesthetics (3 items), information quality (4 items), and the mHealth app’s subjective quality (4 items) [36]. Each question is rated against 5 different response options, which are later summed and averaged to generate a mean score for the individual subscales. Mean scores range from 1 to 5, with higher scores denoting better quality in the particular domain represented by the subscale. This tool has been used to evaluate mHealth apps on well-being [37] and mindfulness [38], and it has been found to possess high internal consistency (Cronbach α=.90) and test-retest reliability (intraclass correlation coefficient range 0.66-0.70). Additionally, it is pitched at a readability level of those aged 12 to 13 years, which also renders it suitable for use among elderly participants who are less educated.

Prior to study initiation, the team sought and received approval from the local ethics board (National Healthcare Group Domain-Specific Review Board [2019/01002]). Written informed consent was obtained from all enrolled participants, and the respective institutions’ personal data protection act will be strictly adhered to during the trial period.

## Results

### The iCanManage Mobile App

Our eventual product was the iCanManage mobile app on the platform of BuddyCare, which has three special features: a surgical timeline, search functionality, and a menu tab. The timeline stretches over a 29-day perioperative phase (14 days before surgery, the day of surgery, and 14 days after surgery) where users will receive information packages listing important tasks about how to prepare for surgery, postsurgery monitoring, and discharge care on a daily basis. In addition, users will also be introduced to mindfulness-based practices, which are purposed to facilitate emotional coping and regulation. Examples of such include mindful breathing, body scan, loving-kindness meditation, and mindful coping. Daily tasks are color coded based on the urgency for review and completion, and reminders are sent at scheduled timings to ensure tasks are acknowledged before they become overdue. Positive slogans are also refreshed periodically to motivate users to maintain a positive outlook or comfort them in their setbacks.

The search functionality enables participants to locate and retrieve information within the timeline easily by entering keywords. This is necessary given the vast amount of text, files, and multimedia materials embedded in the app, which may only become relevant for the user at specific time points of the treatment continuum. Users may also revisit information retrospectively or prospectively as they wish.

Under the menu tab, users will find peer support videos, ostomy care videos, and help hotlines. Through the messaging feature, users may also communicate their concerns to the attending care team and seek tailored advice. Pictures of wound and stoma conditions can be sent anonymously, and remote surveillance and consultation can be conducted. Through this digital solution, we hope users will be more equipped, confident, and independent when undergoing elective CRC surgery. During the trial period, the participants randomized to the intervention arm will be given an activation code that permits personalized access to the digital care path within the mobile app installed on their handheld devices. By doing so, individuals may feel more invested in their own treatment progress and work toward their set goals. An overview of the components and content is outlined in Multimedia Appendices 2 and 3, while screenshots of the developed solution are shown in Multimedia Appendix 4 (image A: English version; image B: Chinese version).

### Preliminary Findings on the Quality of BuddyCare

The uMARS survey data from 5 patients with CRC were analyzed descriptively. Among these participants, 4 were females and 1 was male. The mean age of these participants was 57.8 years old (SD 3.77) (range: 54 to 64 years old). The mean subscale scores were 2.8/5 (SD 0.40) for engagement, 3.9/5 (SD 0.34) for functionality, 3.5/5 (SD 0.38) for aesthetics, 3.4/5 (SD 0.34) for information quality, and 2.4/5 (SD 0.42) for app subjective quality. Besides these 5 subscales, an additional component of the uMARS known as perceived impact (consisting of 6 items) rated from “1=strongly disagree” to “5=strongly agree” was measured. The mean score for perceived impact was 4 out of 5, and the overall app quality mean score was tabulated to be 3.4 out of 5. Moreover, 2 participants provided additional comments in the empty field of the questionnaire; 1 of them stated that some of the contents within the app were repetitive, while the other said it was good to try out a new program, and that it was a well-done initiative with room for improvement.

### Strategies to Overcome Potential Limitations

Although much has been done to increase the usability and acceptability of the digital solution, there remain several potential limitations. For instance, elderly users who are not technology savvy may encounter difficulties when navigating the mobile app and, as a result, become more confused and distressed. Once a scenario as such occurs, users tend to become hesitant or resistant to subsequent health technologies. Moreover, poor eHealth literacy may further hinder their understanding of the presented information. Given the high incidence rates proportionate to the age of individuals diagnosed with CRC in the local context [39], we propose the inclusion of family caregivers during the trial to avoid underutilization of the app and its benefits being diminished. Study team members will provide face-to-face training on the day of recruitment to introduce all features, functions, and materials within the app. An instruction manual will be provided at the end of the training session for reference should any technical issues render the user unable to return to the app’s main page. Moreover, telephone calls will be made by academic researchers weekly to ensure the app is operating properly. Lastly, all contents were translated and will be made available in simplified Chinese language to suit the linguistic demographics of the local population. That being said, our multicenter trial will recruit both English- and Chinese-speaking patients scheduled to undergo elective CRC surgeries.

## Discussion

### Principal Findings

Telemedicine has been around for some time, but it was not until lately that a growing interest in interactive health communication technologies has been observed among users of health care and their providers [40]. As the world continues to battle COVID-19 and countries strive to embrace smart technologies, there is a compelling need for more initiatives to consistently lead seniors toward the adoption of digitalization, and our trial paved the way for such opportunities. In this regard, our multidisciplinary international collaboration also unveiled the following valuable insights.

Firstly, as with most studies, researchers are inclined to focus only on raising the technical skill level of the users, often forgetting that health care providers themselves ought to be knowledgeable and proficient in the said technology [41]. Cancer care is complex and requires multimodal treatment over prolonged periods of time. As mentioned earlier, disease management and self-care for patients with CRC can be exceptionally trying due to altered bowel patterns, dietary intolerance, and stoma-related concerns [42]. Multiple points of contact with various allied health care professionals are common and necessary, yet this would mean frequent hospital visits. Our collaboration is hopeful that health technologies will strengthen the delivery of holistic care through better integration of medical data, standardization of educational counseling, and increased patient autonomy. To achieve this, barriers prohibiting health care professionals from actual utilization, such as shortage of easily accessible and high connectivity computers, security issues, and the lack of staff training, should be eliminated [43].

Secondly, much controversy has been associated with web-based or online psychotherapies. The use of technology is generally perceived to impede the interpersonal therapeutic relationship between the psychologist and the patient, thereby reducing treatment effectiveness [44]. However, input from our multidisciplinary teammates ushered possibilities of transforming traditional, formal psychotherapy into informal practices that can be applied or exercised in the individual’s daily life. While such options offer greater flexibility [45], careful attention should be paid to maintain open channels for communication and assessment of patients’ responses, as well as to safeguard the confidentiality of information shared over the internet platform being used, which will be further explored in our trial.

Lastly, preliminary uMARS data from the actualization of our digital solution raised several points for reflection and future consideration. Although these findings cannot be generalized to the entire CRC population, scores of each subscale provided a glimpse of the participants’ preferences and helped gauge how well the digital solution addressed their needs. According to the results, high levels of functionality, aesthetics, information quality, and perceived impact indicated that the BuddyCare mobile app is generally usable, pleasant to look at, credible, and relevant for our participants’ medical condition. Conversely, moderate levels of engagement revealed that our digital solution was still lacking in interactivity and customization. This could mean that participants desire an exchange of information between themselves and the software, researchers, or clinicians, even though the enablement of such feature may increase the technical complexity of the app. In terms of subjective quality, lower-than-midpoint values suggest that the app was likely not convincing enough to warrant a recommendation to others or payment for its usage in the long run. This is reasonable given the relatively young age of these 5 participants who reported the data. The young, being more exposed to digital technologies, may have higher expectations when evaluating new mobile apps. Moreover, our findings agree with some of the consolidated themes derived from stakeholder perceptions delineated in a study by Mercer and colleagues [46]. These include the need to explore flexibility in design to achieve sustained usage, as well as to leverage the knowledge of behavioral psychology to improve user engagement. Considering these pertinent issues, more data from the full-scale randomized controlled trial will be necessary to verify and support these findings before any change is to be made to the digital solution.

### Conclusion

This paper detailed the sequential development of the iCanManage smartphone-based psychosocial program to be used in a multicenter randomized controlled trial. This patient-centric digital solution will be the first to deliver a combination of surgery and psychosocial care to patients scheduled to undergo elective CRC surgeries and their family caregivers. If found to be effective, the iCanManage will see increased adherence to prescribed treatments, increased patient satisfaction through better health outcomes, and smoother transitions from hospital to home following surgery. In the long run, better allocation of personnel, time, and costs can be attained, and the economic burden can be significantly reduced when extended to elective surgeries for other disease types.

## Appendix

Multimedia Appendix 1 Theory-guided contents.

Multimedia Appendix 2 Overview of the components and contents within the iCanManage mobile app.

Multimedia Appendix 3 Daily outline of contents.

Multimedia Appendix 4 Screenshot images of the completed digital solution in English and Chinese versions.

## Abbreviations

CRC: colorectal cancer
uMARS: Mobile App Rating Scale, user version
