# Supplementary material for: A Theory-Based, Multidisciplinary Approach to Cocreate a Patient-Centric Digital Solution to Enhance Perioperative Health Outcomes Among Colorectal Cancer Patients and Their Family Caregivers: Development and Evaluation Study
Source: J Med Internet Res. 2021 Dec 7;23(12):e31917. doi: 10.2196/31917 (PMC8693179; doi:10.2196/31917)
Supplement: Multimedia Appendix 1 [file jmir_v23i12e31917_app1.docx]

**Appendix 1**: Theory-guided contents.

| Objectives and outcomes | | | | | Contents of the iCanManage program |
| --- | --- | --- | --- | --- | --- |
| **Self-efficacy** | | | | | |
|  | Mastery experience | Instill self-efficacy through information provision, practice, overcoming obstacles, and goal setting | | - Educational materials in the form of text, audios, and videos. Information about colorectal cancer, surgery, adjuvant therapy (eg, chemotherapy and radiation therapy), symptom management, and stoma care will be embedded in the app to strengthen participants’ preparedness to manage a task and challenge or perform a skill. - Goal setting and self-monitoring logs within the app will be used to promote the development of small, incremental goals. | |
|  | Vicarious experience | Encourage learning from successful others to achieve effective coping | | - Videos on colorectal cancer survivors who will share their successful approaches to coping and strategies to enhance the adaptation process. | |
|  | Verbal persuasion | Provide positive reinforcement | | - Positive slogans that acknowledge the effort that has been invested and motivation for continuation of positive actions and attitudes. - Chat room function for social interaction and exchange of words of encouragement, which will help reassure, empathize, and motivate the need for continual effort. Through communication, health care professionals who show appreciation to the participant will also enhance the participants’ self-efficacy and coping abilities. - Telephone calls from research team members who will listen to the participants’ struggles, empathize, and convey positive appraisals that will boost the participants’ morale. | |
|  | Physical and affective states | | Enable the regulation of physical reactions and emotional proclivities | - Mindfulness-based practices that will enable the participants to eliminate or reduce stress and anxiety through imparting skills such as monotasking and developing awareness to the physical, emotional, and cognitive state of the individual. - Positive psychology reading materials that will help eradicate cognitive distortions, negative emotions and unhealthy behaviours. By the use of such materials, pitfalls in thought processing can be replaced with optimism, focus directed to strengths instead of weaknesses, and thereby improve their reactions toward stress, tension and uplift their moods. - Information on myths and misconceptions surrounding colorectal cancer, treatment and stoma care. | |
| Psychological well-being | | | | Improve participants’ psychological well-being through raising self-efficacy levels and provision of social support in various forms. | |
| Social support | | | | - Peer emotional support from videos of colorectal cancer survivors who show appreciation toward their family caregivers - Professional informational support from chat room function - Institutional-based tangible support through the provision of help hotlines - Affectionate support and positive social interaction from telephone calls by research team members | |
| Quality of life | | | | Participant’s quality of life will be enhanced through improvements in self-efficacy, psychological well-being and levels of social support availability. | |
