# Supplementary material for: A Theory-Based, Multidisciplinary Approach to Cocreate a Patient-Centric Digital Solution to Enhance Perioperative Health Outcomes Among Colorectal Cancer Patients and Their Family Caregivers: Development and Evaluation Study
Source: J Med Internet Res. 2021 Dec 7;23(12):e31917. doi: 10.2196/31917 (PMC8693179; doi:10.2196/31917)
Supplement: Multimedia Appendix 2 [file jmir_v23i12e31917_app2.docx]

**Appendix 2**: Overview of the components and contents within the iCanManage mobile app.

iCanManage

CRC^a^ surgery-related information:

- Introduction to colorectal cancer
- Surgery and stoma formation procedures
- How to prepare for surgery (instructions on fasting, bowel preparation, carbohydrate loading, and nutritional supplements)
- Hospitalization and discharge planning
- Postdischarge care and monitoring
- Follow-up visits

Patient education materials:

- Nutrition or diet: practical tips for general CRC and stoma patients
- ADLs^b^ or physical activity or going back to work or Muslim ostomate
- Coping with diarrhea or constipation
- Chemotherapy and radiotherapy
- Caregivers’ hub and coping strategies

Ostomy care:

- Step-by-step guide on how to prepare, apply, empty, and remove ostomy appliance
- Practical tips on diet, travel, and sex

Psycho-education:

Using principles of positive psychology and mindfulness:

- To enhance coping with negative feelings (eg, anxiety, depression, fear, and uncertainty)
- To overcome social stigma and isolation
- To increase self-efficacy regarding the symptom management
- Reinforce adaptive and helpful behaviors

Help resources:

- Frequently asked questions
- Help hotlines
- Chat with your care team (messenger)

Peer support resources:

- CRC survivors’ successful stories
- Hospital-based peer-sharing sessions and events
- External support group sessions and activities

Information

Support

^a^colorectal cancer.

^b^ADLs: activities of daily living.
