# Supplementary material for: A Theory-Based, Multidisciplinary Approach to Cocreate a Patient-Centric Digital Solution to Enhance Perioperative Health Outcomes Among Colorectal Cancer Patients and Their Family Caregivers: Development and Evaluation Study
Source: J Med Internet Res. 2021 Dec 7;23(12):e31917. doi: 10.2196/31917 (PMC8693179; doi:10.2196/31917)
Supplement: Multimedia Appendix 3 [file jmir_v23i12e31917_app3.docx]

**Appendix 3**: Daily outline of contents.

| Perioperative periods | | Table of contents | Media materials |
| --- | --- | --- | --- |
| **Before surgery** | | | |
|  | Day 1 | What is colorectal cancer? | - (Video) hospital admission process |
|  | Day 2 | What is mindfulness? | - (Video) leaves swaying |
|  | Day 3 | Myths and benefits of mindfulness | - (Image) myths and benefits of mindfulness |
|  | Day 4 | Let’s try monotasking | - (Video) origami handicraft activity |
|  | Day 5 | Developing a sense of awareness | - Journaling using 5 senses - (Audio) mindfully eating a fruit practice |
|  | Day 6 | Preparedness | - (Image) preparedness |
|  | Day 7 | Open monitoring | - (Audio) doing the body scan |
|  | Day 8 | Mindful breathing | - (Audio) breathing exercise |
|  | Day 9 | Muscle relaxation | - (Audio) relaxation exercise |
|  | Day 10 | Mindfulness of abilities | - (Text) identify strengths and weaknesses |
|  | Day 11 | Mindfulness of thoughts and emotions | - (Video) game on lining the dots - (Image) thinking errors |
|  | Day 12 | Taking mindful actions to take care of ourselves | - (Image) what is resilience? - (Text) how to build resilience |
|  | Day 13 | Resistance vs acceptance | - (Video) game on maze |
|  | Day 14 | Mindful breathing | - (Audio) breathing exercise |
| Day of Surgery | | Mindful breathing | - (Audio) breathing exercise |
| **Postoperative** | | | |
|  | Day 1 | Openness: option A or B? does it matter? | - (Video) Louis’ story |
|  | Day 2 | Muscle relaxation | - (Audio) relaxation exercise |
|  | Day 3 | Loving-kindness meditation | - (Audio and image) loving-kindness practice |
|  | Day 4 | Transience: all things will pass | - (Audio) storytelling |
|  | Day 5 | Gratitude and its benefits | - (Image) benefits of adopting an attitude of gratitude |
|  | Day 6 | Growing happiness | - (Audio) how to grow happiness |
|  | Day 7 | Mindful sitting | - (Audio) sitting meditation exercise |
|  | Day 8 | Open monitoring | - (Audio) doing the body scan |
|  | Day 9 | Mindful coping: who am I? | - (Audio) finding self-identity exercise - (Image) what shapes one’s identity |
|  | Day 10 | Improving sleep quality | - (Audio) sleeping practice |
|  | Day 11 | Reducing anticipatory symptoms (eg, nausea) | - (Audio) thought-stopping, cognitive restructuring exercise |
|  | Day 12 | Mindful choice | - (Image) how choices in life are made |
|  | Day 13 | Moving beyond cancer | - (Audio) relaxation exercise - (Text) poem reading |
|  | Day 14 | Summary of activities | - Goal setting - Journaling - (Image) ways to practice mindfulness in everyday life |
