# Supplementary material for: A Theory-Based, Multidisciplinary Approach to Cocreate a Patient-Centric Digital Solution to Enhance Perioperative Health Outcomes Among Colorectal Cancer Patients and Their Family Caregivers: Development and Evaluation Study
Source: J Med Internet Res. 2021 Dec 7;23(12):e31917. doi: 10.2196/31917 (PMC8693179; doi:10.2196/31917)
Supplement: Multimedia Appendix 4 [file jmir_v23i12e31917_app4.docx]

**Appendix 4:** Screenshot images of the completed digital solution in English and Chinese versions.


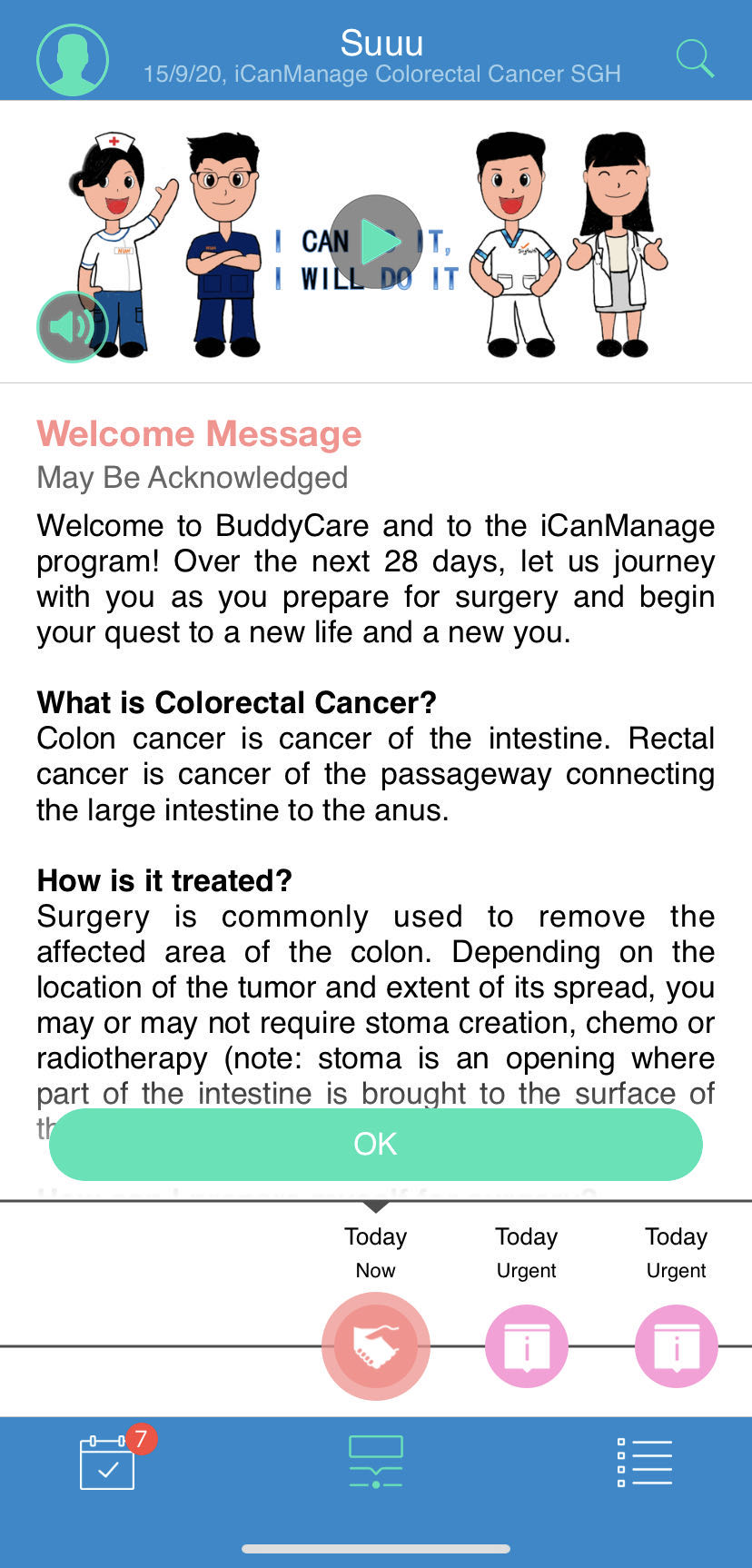
A

Menu

Participant code

Participant code


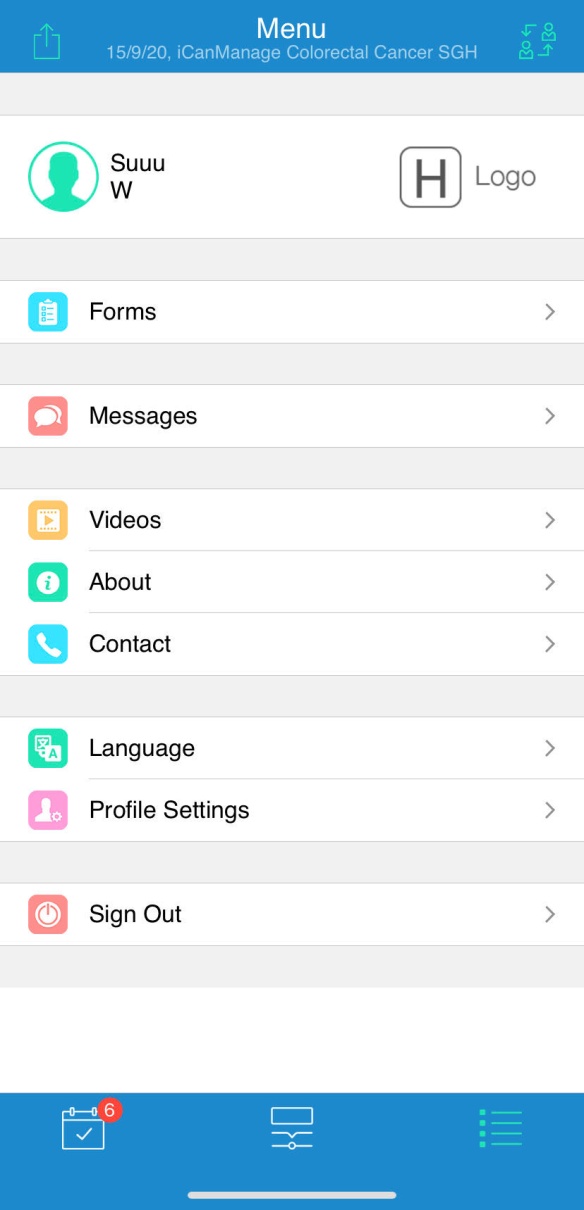

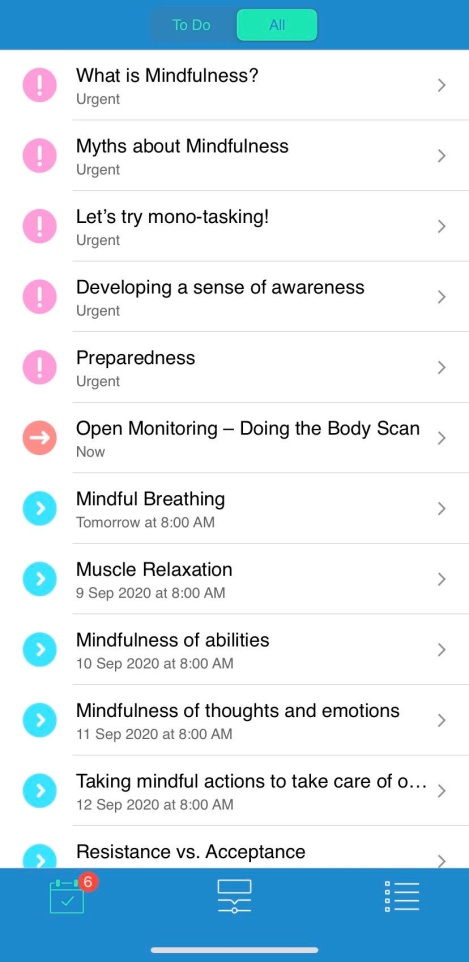

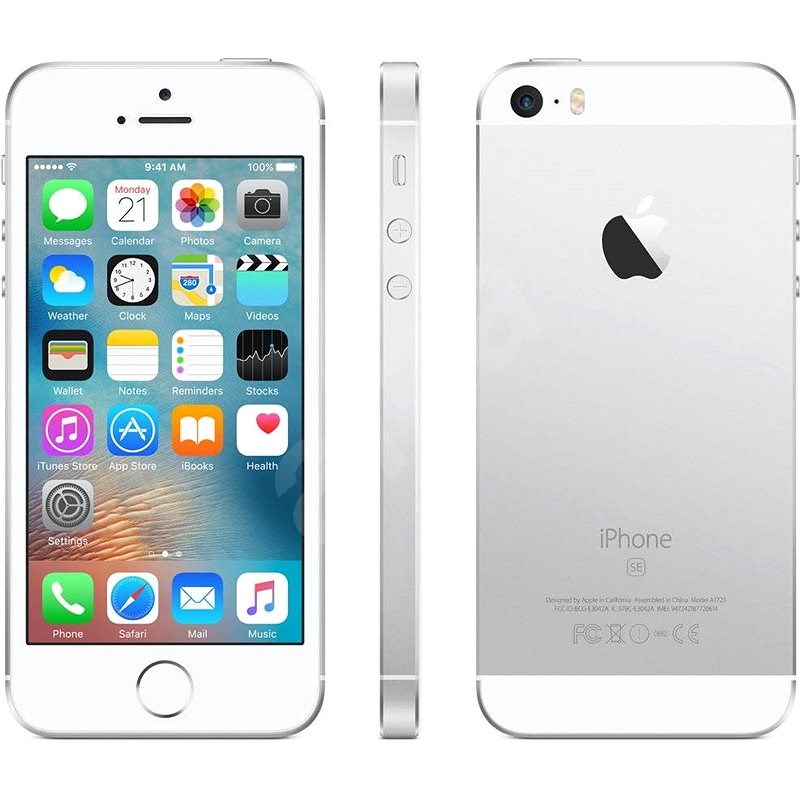


B


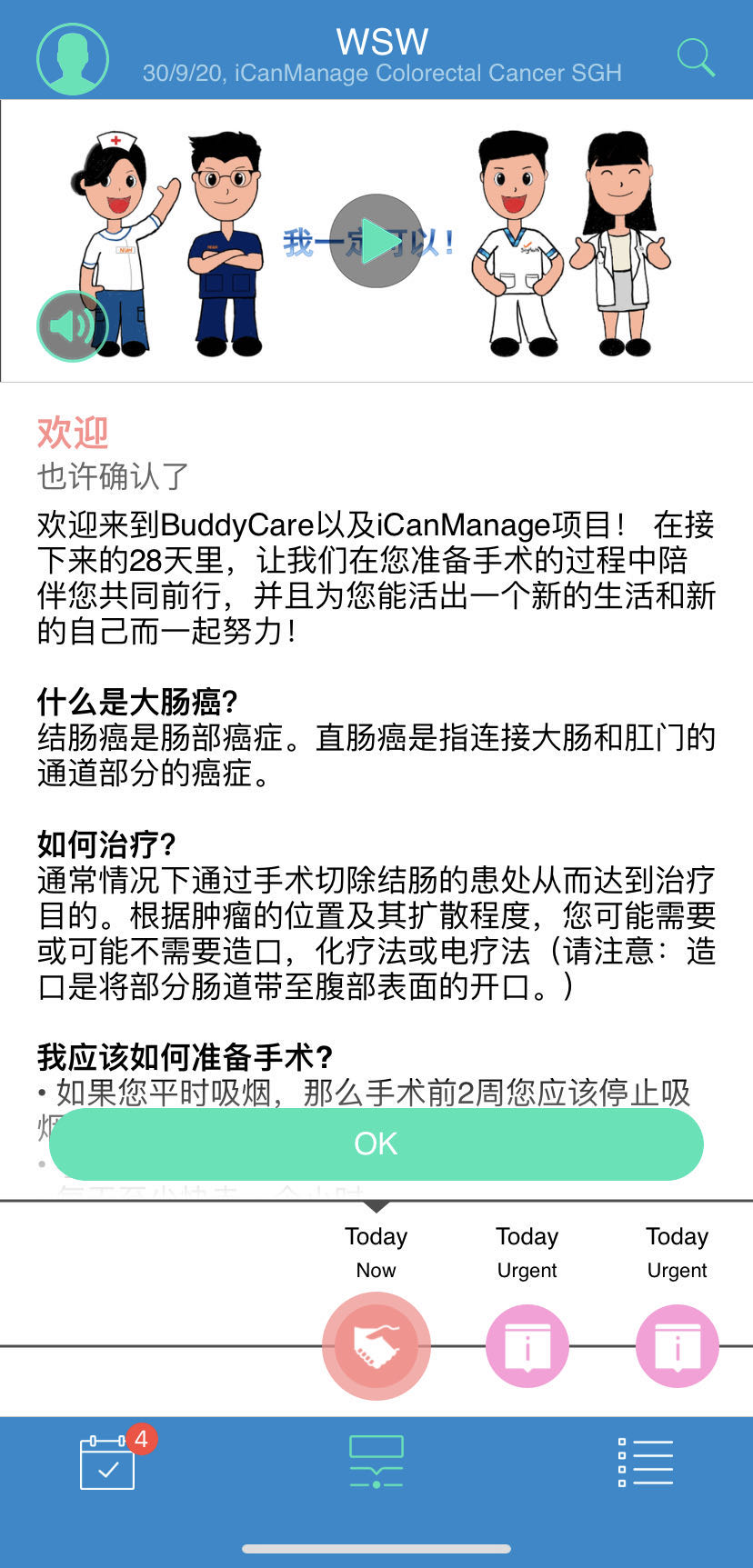

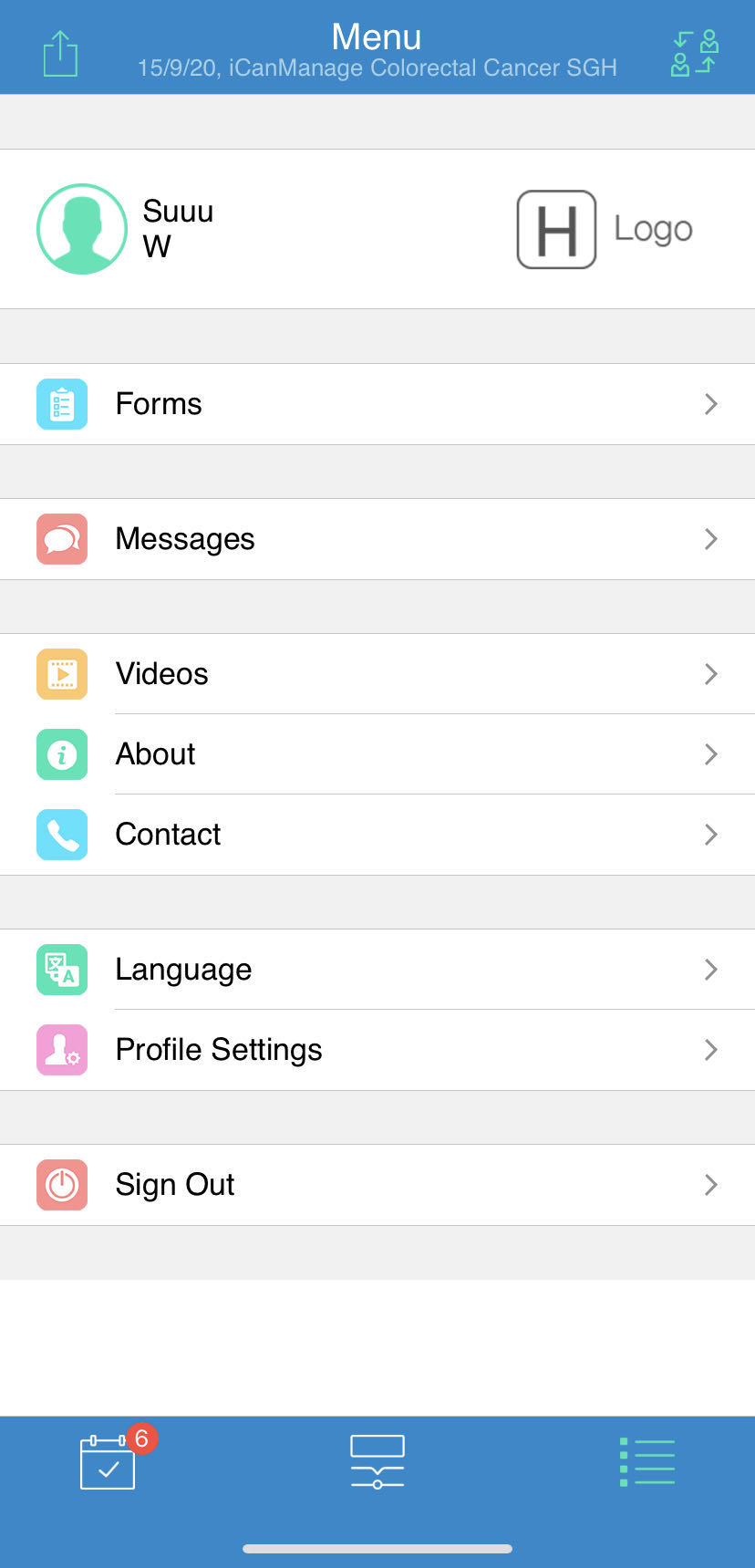

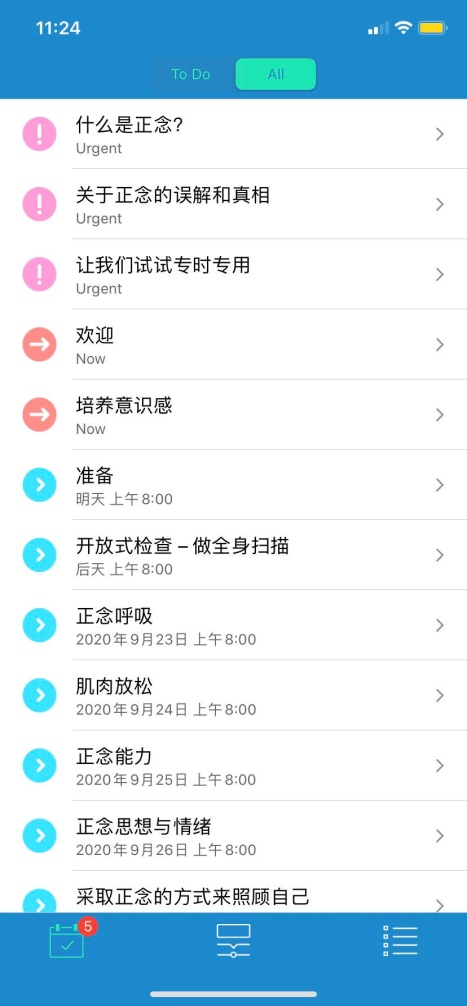

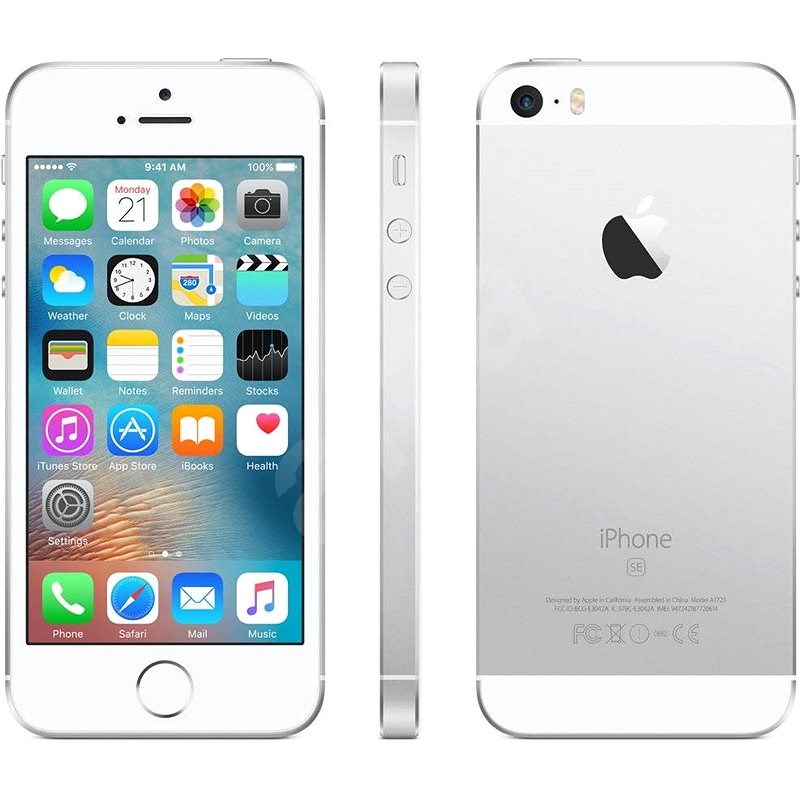


用户编号

目录

（填写表格）

（简讯 / 讯息）

（视频）

（关于）

（联络 / 热线）

（个人资料设置）

（语言 – 英语 / 华语）

（登出）

用户编号
